# Health Tract No. 318

**Published:** 1868-10

**Authors:** 


HEALTH TRACT No. 318.

### RECOGNITION HEREAFTER.

Whatever sentiment of the mind is universal, may well be considered as an inherent quality inseparable from it, a part of it, and created with it, hence, of Divine Origin; else, why should Divinity, who is the very embodiment of Benevolence, incorporate with every soul brought into existence, as a part of it, a false sentiment, or belief, or instinct.

All nations of all ages believe in a hereafter, and there is a hereafter. All nations of all ages believe in a future state of rewards and punishments, and there is a future state of rewards and punishments.

All nations of all ages have a sense of remorse for the perpetration of what is considered as a wrong, hence there is a conscience, which bears testimony against wrong doing, as wrong.

All nations of all ages have consoled themselves in the death of dear ones, that they shall know them lovingly, when they meet in the future world, hence we may infer that there is a blessed recognition hereafter, of all the good, else, how can the Beneficent One allow the creatures of his power, the works of his hands, the children of his love, to universally feed on hopes that can never be realized; to take comfort in anticipations which can have no existence? Will He who is the personification of "Love" itself, thus mock the crushing grief of those whom he made in his own image? How can it be?

"I shall go to him, but he shall not return to me," said King David, when he would comfort himself for the death of his darling child; such a speech would be the merest nonsense, if when David went to him in Heaven, he would not recognize from any other angel children there. Certainly we shall not know less in heaven than we do here; one of the greatest delights in any society or company is the personal knowledge of those who are present. Well may we suppose that next to the thrill of bliss which shall sweep across the soul at the first sight of the blessed Savior in Paradise, will be, the joy unutterable of finding the loved ones of earth there, safe; forever safe! and happy too!

"Friends, e'en in heaven, one happiness would miss,
"Should they not know each other when in bliss."

Memory cannot die, otherwise the soul would lose its identity; because our experiences are a part of ourselves. And it can not be supposed that the terrestrial experiences of the soul shall become a blank in heaven, shall be forever blotted out; for surely it will highten the bliss of immortality, to compare the dangers and toils and sins of earth, with the safety and rest and holiness of heaven!

Paul said to the Hebrew church, the converted Jews, that Angels were the "ministering spirits" waiters, guards, care takers of God's people in this world; their helpers towards heaven; and certainly they will continue to know us when we get there; and will it not highten the gladness of the meeting, that we shall intuitively know them as our friendly attentants in the world we have left far below; just as Peter, James and John, in the Mount of Transfiguration, intuitively knew, without an introduction, that it was Moses and Elias which talked with the Savior. The "sick man" in Tophet recognized Lazarus, and knew Abraham, intuitively, for as soon as he saw him he spoke to him, calling his name.

If there be no recognition hereafter, whence the universal desire and hope and longing to be buried with the departed, an idea so touchingly presented by Mrs. Sigourney of a dying child,

"One only wish she uttered, — While life was ebbing fast;
Sleep by my side, dear mother! — And rise with me at last."

And these things being so, let us cherish the thought when remorseless death crushes our fondest hopes, that we shall meet the good again, not only as a means of inciting us to live more like them, but as a means of joyousness of spirit to give a new sparkle to the life blood as it courses through the veins, carrying with it an energy and healthfulness which shall vitalize the body and give it a new lease of existence.

